# Association between dietary index of gut microbiota and constipation in a female population: a cross-sectional study

**DOI:** 10.3389/fnut.2025.1562258

**Published:** 2025-03-31

**Authors:** Wangfeng Lu, Gang Feng, Lei Liu, Qi Ding

**Affiliations:** ^1^Department of Gastrointestinal Surgery, Shangluo Central Hospital, Shangluo, Shanxi, China; ^2^Department of Anorectal, Shangluo Traditional Chinese Medicine Hospital, Shangluo, Shanxi, China; ^3^Department of Pharmacy, Shangluo Central Hospital, Shangluo, Shanxi, China

**Keywords:** female populations, DI-GM, NHANES, constipation, gut microbiota, dietary index

## Abstract

**Objective:**

To examine the potential association between dietary index of gut microbiota (DI-GM) scores and constipation in adult women in the United States.

**Methods:**

This cross-sectional study used data from adult participants in the 2005–2010 US National Health Survey (NHANES). The missing values in the covariables were filled by multiple interpolation. Multivariate logistic regression models were used to determine the odds ratios (OR) and 95% confidence intervals (CI) for the association between DI-GM and constipation. Subgroup analyses were also performed to examine the possible interactions between DI-GM and constipation.

**Results:**

Of the 7,325 subjects, 887 reported constipations, with a prevalence of 12.1%. After adjustment for multivariate modeling, the DI-GM score was significantly associated with constipation (0.92 [95% CI 0.87–0.96]; *p* = 0.001). Similar results were found for the association of beneficial gut microbiota score with constipation (OR 0.89 [95% CI 0.84 to 0.95]; *p* = 0. 001). Subgroup analyses revealed that the relationship between DI-GM scores and constipation remained stable (*p* > 0.05).

**Conclusion:**

DI-GM was negatively associated with the incidence of constipation in the female population. Clinicians should consider the influence of dietary structure on the treatment of constipation in women. Dietary intervention can be an important strategy for the comprehensive treatment of constipation.

## 1 Introduction

Constipation is a chronic disease characterized by difficulty in defecation and a decrease in the number of bowel movements (1). The prevalence of constipation in the general population ranges from 3 to 27% (2), and more attention has been paid to constipation in children and the elderly population in the course of clinical treatment (3, 4). In recent years, with the increase of life pressure borne by women in modern society, the incidence of constipation is on the rise (5, 6), which seriously affects the daily life of the female population and brings a huge medical burden to economic and social development (7).

Dietary factors are often cited as the main cause of constipation, and dietary modifications often influence changes in the intestinal microflora (8). Dietary index of gut microbiota (DI-GM) is an assessment index to evaluate the relationship between gut microbiota and dietary factors. Developed by Kase et al. based on a large body of research literature, it is a dietary pattern that effectively identifies beneficial or unfavorable gut microbiota (9). For example, the consumption of whole grains and bran increases the levels of *Bifidobacterium* spp. and *Lactobacillus* spp., which are beneficial to the gut flora, whereas the consumption of red meat-rich foods increases the levels of *Ruminococcus, Alistipes, Blautia*, and *Bilophila* genera, which are unfavorable to the gut flora (10). Categorized according to whether a food component has a positive or negative effect on the gut microbiota, and is used to assess the quality of diets associated with the maintenance of normal gut flora (11).

Gut microbiota dysbiosis not only interferes with microbially mediated gut secretion and metabolic dysfunction, leading to constipation, but also interferes with the modulation of bowel movements by the brain-gut-microbe axis (12, 13). The number and distribution of intestinal flora play a very important role in maintaining intestinal function, and increasing the number of beneficial intestinal microbiota is commonly used to treat constipation (14). In recent years, simpler and more effective ways to improve intestinal flora and relieve constipation have been explored, and much attention has been paid to modifying the structure of the intestinal flora by adjusting dietary patterns (15–17). However, among the reported studies on constipation, little is known about the relationship between DI-GM and constipation in female populations. In this study, we examined the relationship between DI-GM and constipation in a female population in the United States using data from the NHANES database. We hypothesized that there would be an association between DI-GM scores and constipation, with higher DI-GM scores being associated with a lower risk of constipation.

## 2 Materials and methods

### 2.1 Data sources

The NHANES is a nationally representative survey of the health and nutritional status of the non-institutionalized population of the United States, using stratified, multistage probability cluster sampling (18). Data on constipation were only available for the 2005–2010 NHANES cycles. The NHANES study protocol was approved by the NCHS Research Ethics Review Board. Participants provided written informed consent at enrolment (19). The study conducted at Shang Luo Central Hospital (Shang Luo, China) was deemed exempt by the institutional review board because of the use of publicly available anonymized data. This study adhered to the Strengthening of the Reporting of Observational Studies in Epidemiology (STROBE) reporting guidelines.

### 2.2 Study design and population

**Figure 1:**
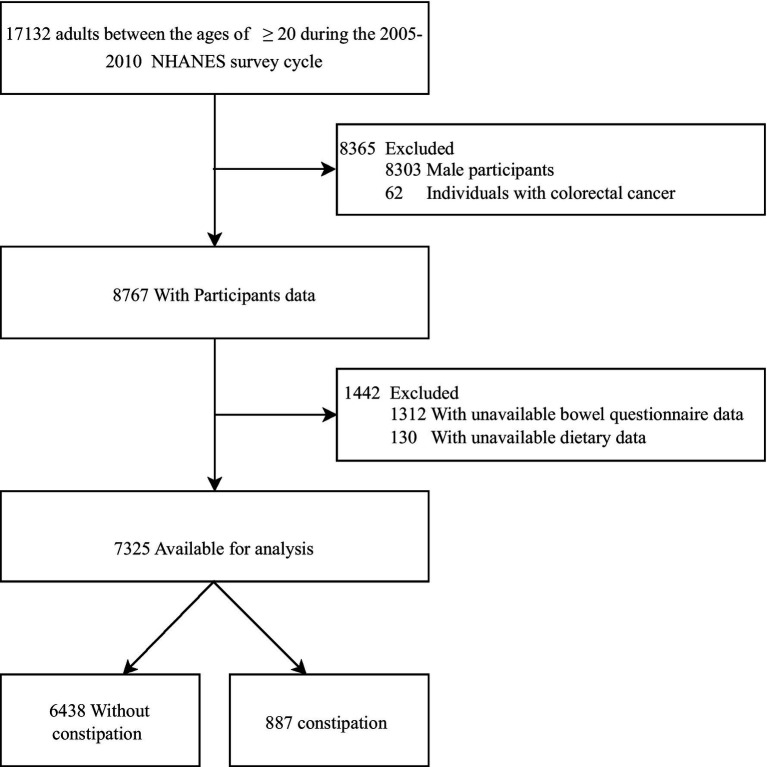
Flow chart of the screening of the NHANES 2005–2010 participants.

This study collected data from the US National Health Survey (NHANES; 2005–2010). The following exclusion criteria were used to limit the analysis to patients with constipation between the ages of ≥20 years: colorectal cancer, missing data from the bowel questionnaire, missing/unavailable gut microbiota diet, and missing data for other covariates were interpolated using multiple interpolations. Figure 1 shows the flowchart of the subject recruitment process.

### 2.3 Ascertainment of DI-GM and constipation

Constipation was assessed using the NHANES Bowel Health Questionnaire, based on the texture of the stools and the frequency of bowel movements. The questionnaire asked the participants to rate the texture of their stools and the frequency of their bowel movements. The Bristol Stool Frequency Scale (BSFS) consists of cards with different colored pictures and explanations of seven stool types to measure the consistency of their stools (20). Participants were asked to choose the number closest to the type of stool they usually see. BSFS type 1 (characterized by hard, nutty lumps) or type 2 (sausage-like, but lumpy) was used to diagnose constipation. BSFS types 3 (smooth and soft, such as a sausage or snake), 4 (smooth and soft), and 5 (soft plaques with sharp edges) are used to diagnose normal bowel function. BSFS types 6 (consisting of fluffy crumbs with rough edges and a pasty texture) or 7 (watery with no solid crumbs) were used to diagnose diarrhea. Fewer than three bowel movements per week were classified as constipation, between three and 21 bowel movements per week as normal, and more than 21 bowel movements per week as diarrhea. In this study, participants with stool types I and II and fewer than three bowel movements were classified as constipated and the others as non-constipated (21, 22).

Fourteen foods or nutrients were identified as components of DI-GM in the NHANES database based on the scoring criteria in an article by Zheng et al. (23). DI-GM included avocado, broccoli, chickpeas, coffee, cranberries, fermented dairy products, cottage cheese, green tea (because it was not available in NHANES), soy, and whole grains as beneficial components. In contrast, red meat, processed meat, refined grains, and high-fat diets (approximately 40% energy from fat) are considered harmful. NHANES data from 2005 to 2010 were used for dietary assessment calculations. The methodology for calculating DI-GM, its components, and scoring criteria can be found in the NHANES 2005–2010 data. For beneficial to gut microbiota items, the item was scored as 1 if consumption of the item was ≥ the gender-specific median, and 0 otherwise. For unfavorable gut microbiota items, the item was scored as 0 if consumption was ≥ the sex-specific median or 40% (high fat) and 1 otherwise. The scores were summed to give a total DI-GM score, which ranged from 0 to 13 (including beneficial to gut microbiota [range 0 to 9] and unfavorable to gut microbiota [range 0 to 4]) and was scored on a scale of 0–3, 4, 5, and 6 (24).

### 2.4 Covariates

Based on previous NHANES research, potential covariates included in the analyses were age, sex, race/ethnicity (categorized as non-Hispanic White, non-Hispanic Black, Mexican American, or other), marital status (categorized as married/cohabitating or single, including never married, separated, widowed, or unmarried), including never married, separated, divorced, or widowed, years of education (less than 9, 9–12, or more than 12), and three levels of household income based on poverty income: low (PIR ≤ 1. 3), medium (PIR, 1.3–3.5), and high (PIR > 3.5) (25). Body mass index (weight (kg)/height (m^2^), height and weight measured at a mobile health screening center), and physical activity are defined as ‘organized or unorganized sports, fitness or recreational activities (e.g., gym work, cycling, running and all team sports), active travel (e.g., walking or cycling), and any other physical activity in, at or around the workplace, at home or any other physical activity while volunteering’. Physical inactivity was defined as less than 150 min of moderate-intensity physical activity per week (26). Smoking was defined as having smoked ≥100 cigarettes in a lifetime, alcohol consumption as at least 12 drinks per year, hypertension (physician-diagnosed systolic blood pressure ≥ 140 mmHg or diastolic blood pressure ≥ 90 mmHg or taking antihypertensive medication) (27), stroke (physician-diagnosed) and coronary heart disease (physician-diagnosed). Fasting blood glucose level ≥ 7.0 mmol/L, blood glucose level ≥ 11.1 mmol/L in a 2-h randomized oral glucose tolerance test, or use of diabetes medication/insulin for diagnosis of diabetes (28). Carbohydrate and energy intake was obtained by asking respondents to recall all beverages consumed and all foods eaten in the 24 h before the interview. The U.S. Department of Agriculture Nutrient Database was used to calculate data on dietary nutrient intake (29). The use of antidepressants was classified as ‘yes’ or ‘no’ based on the report of the participant (30).

### 2.5 Statistical analysis

Continuous variables are expressed as means and corresponding 95% confidence intervals (CIs), and categorical variables are expressed as percentages and 95% CIs. Normally distributed data were analyzed using a one-way analysis of variance (ANOVA), and skewed data were analyzed using the Kruskal-Wallis test. Categorical variables are expressed as proportions (%), and continuous variables are expressed as mean (standard deviation [SD]) or median (interquartile range [IQR]), as appropriate. Differences between groups were assessed using one-way ANOVA (for normally distributed data), the Kruskal-Wallis test (for skewed data), and the chi-squared test (for categorical variables). Multivariate logistic regression models were used to determine the odds ratios (OR) and corresponding 95% CIs for the association between the DI-GM scores and constipation. Model 1 was adjusted for sociodemographic characteristics (age, sex, marital status, race/ethnicity, education, household income, physical activity, body mass index (BMI), smoking status, and alcohol consumption status). Model 2 was adjusted for the factors in Model 1 plus hypertension, cardiovascular disease (CVD), stroke, diabetes, antidepressant use. Model 3 was adjusted for the factors in Model 2 plus energy intake, and carbohydrate intake. To assess the stability of the relationship between DI-GM scores and constipation in the population, multiple imputation by chained equations (MICE) and repeated main analyses. To account for missing baseline data, we used multiple imputation based on 5 imputed datasets. and subgroup analyses were performed according to age, physical activity, body mass index, and diabetes status. Heterogeneity and interactions between subgroups were assessed using logistic regression models and likelihood ratio tests. Statistical power was not calculated *a priori* as the sample size was based entirely on the available data. Analyses were performed using R (version 4.2.1; R Foundation for Statistical Computing, Vienna, Austria)^1^ and Free Statistical Software (version 2.0; Beijing Free Clinical Medical Technology). In all analyses, a two-sided *p* < 0.05 was considered a statistically significant difference.

## 3 Results

### 3.1 Study population

A total of 17,132 US adults aged ≥20 years, exclusions included: male participants (8303); patients with colorectal cancer (*n* = 62); missing Bowel Health Questionnaire data (*n* = 1,312); missing dietary data (*n* = 130). Consequently, 7,325 subjects were included in the final analysis (Figure 1).

### 3.2 Baseline characteristics

Table 1 summarizes the baseline characteristics of study participants. Of the 7,235 female participants, 887 (12.1%) were diagnosed with chronic constipation, and the mean age (± SD) of the subjects was 48.7 (± 18.0) years. The prevalence of chronic constipation was higher among non-Hispanic White participants, married, middle-income, nonsmokers, alcohol drinkers, more educated, those who exercised ≥150 min/week, those who did not have a chronic disease, and those with lower DI-GM.

**Table 1:** General characteristics of the participants from the national health and nutrition examination survey 2005–2010 cycles.

Characteristics	Total	Without constipation	constipation	*p*-value
No.	7325	6438	887	
Age (year), Mean (SD)	48.7 (18.0)	49.2 (17.9)	45.5 (18.6)	< 0.001
Race/ethnicity, n (%)				0.001
Non-Hispanic White	3550 (48.5)	3146 (48.9)	404 (45.5)	
Non-Hispanic Black	1450 (19.8)	1242 (19.3)	208 (23.4)	
Mexican American	1364 (18.6)	1223 (19.0)	141 (15.9)	
Others	961 (13.1)	827 (12.8)	134 (15.1)	
Marital status, n (%)				0.006
Married	3556 (48.5)	3155 (49)	401 (45.2)	
Never married	1167 (15.9)	1001 (15.5)	166 (18.7)	
Living with partner	543 (7.4)	461 (7.2)	82 (9.2)	
Others	2059 (28.1)	1821 (28.3)	238 (26.8)	
Family income, n (%)				< 0.001
≤1.30	2339 (31.9)	2014 (31.3)	325 (36.6)	
1.31–3.50	2801 (38.2)	2448 (38.0)	353 (39.8)	
>3.50	2185 (29.8)	1976 (30.7)	209 (23.6)	
Educational level (year), n (%)				< 0.001
<9	1993 (27.2)	1737 (27.0)	256 (28.9)	
9–12	1714 (23.4)	1471 (22.8)	243 (27.4)	
>12	3618 (49.4)	3230 (50.2)	388 (43.7)	
Smoking, n (%)				0.045
No	4490 (61.3)	3919 (60.9)	571 (64.4)	
Yes	2835 (38.7)	2519 (39.1)	316 (35.6)	
Drinking, n (%)				0.006
No	1462 (20.0)	1254 (19.5)	208 (23.4)	
Yes	5863 (80.0)	5184 (80.5)	679 (76.6)	
Physical activity, n (%)				0.755
<150 min/week	2534 (34.6)	2223 (34.5)	311 (35.1)	
≥150 min/week	4791 (65.4)	4215 (65.5)	576 (64.9)	
BMI(kg/m^2^), n (%)	29.4 (7.4)	29.6 (7.4)	28.3 (7.0)	< 0.001
Hypertension, n (%)	2955 (40.3)	2641 (41.0)	314 (35.4)	0.001
CVD, n (%)	658 (9.0)	568 (8.8)	90 (10.1)	0.196
Stroke, n (%)	273 (3.7)	234 (3.6)	39 (4.4)	0.261
Diabetes, n (%)	1233 (16.8)	1100 (17.1)	133 (15.0)	0.119
Antidepressants, n (%)	957 (13.1)	836 (13.0)	121 (13.6)	0.587
Energy (kcal/d), Mean (SD)	1769.6 (732.5)	1774.5 (732.9)	1734.2 (729.2)	0.125
Carbohydrate (g/d), Mean (SD)	223.0 (99.8)	222.8 (99.9)	224.2 (99.5)	0.700
DI-GM, Mean (SD)	4.6 (1.6)	4.6 (1.6)	4.4 (1.5)	< 0.001
DI-GM, n (%)				< 0.001
0–3	1808 (24.7)	1556 (24.2)	252 (28.4)	
4	1798 (24.5)	1557 (24.2)	241 (27.2)	
5	1743 (23.8)	1539 (23.9)	204 (23)	
≥6	1976 (27.0)	1786 (27.7)	190 (21.4)	
Beneficial to gut microbiota, Mean (SD)	2.2 (1.2)	2.2 (1.2)	2.0 (1.2)	< 0.001
Unfavorable to gut microbiota, Mean (SD)	2.4 (1.0)	2.4 (1.0)	2.4 (1.1)	0.869

BMI, body mass index; CVD, cardiovascular diseases; DI-GM, dietary index for gut microbiota. The DI-GM ranges from 0 to 13 (including beneficial to gut microbiota [ranges from 0 to 9] and unfavorable to gut microbiota [ranges from 0 to 4]) and grouped according to 0–3, 4, 5, and ≥ 6. The continuous data were shown as mean (standard deviation, SD), and differences between groups were compared using a T-test, one-way analyses of variance (normal distribution), and Kruskal–Wallis tests (skewed distribution). The categorical data were shown as numbers and percentages [n (%)], and differences between groups were compared using the Chi-squared test.

### 3.3 Association between DI-GM score and constipation

As shown in Table 2, after adjusting for age, race/ethnicity, marital status, education level, household income, smoking status, alcohol consumption, physical activity, BMI, CVD, hypertension, stroke, diabetes mellitus, antidepressant medications, energy intake, and carbohydrate intake, Constipation prevalence decreased by 8% per 1 point increase in DI-GM (0.92 [95% CI 0.87–0.96]; *p* = 0.001). After grouping by DI-GM, compared with the control group, the prevalence of constipation was higher in the DI-GM ≥ 6 group in the unadjusted model (OR 0.66 [95% CI (0.54–0.8)]; *p* < 0. 001); in the adjusted model 1, the DI-GM ≥ 6 group was associated with a prevalence of constipation (OR 0.77 [95% CI 0.62–0.95]; *p* = 0. 014); in adjusted model 2, the DI-GM ≥6 group was associated with the prevalence of constipation (OR 0.77 [95% CI 0.62 to 0.95]; *p* = 0. 017). In adjusted model 3, the DI-GM ≥6 group was associated with the prevalence of constipation (OR 0.72 [95% CI 0.58 to 0.9]; *p* = 0. 003). In addition, the prevalence of constipation was significantly reduced with an increase in beneficial gut microbiota (OR = 0.89 [95% CI 0.84 to 0.95]; *p* = 0. 001), whereas an increase in unfavorable gut microbiota was not associated with the prevalence of constipation (OR 0.94 [95% CI 0.86 to 1.02]; *p* = 0.112).

**Table 2:** Relationship between DI-GM and constipation among US adult women participants in NHANES 2005–2010.

	No.	Crude OR (95%CI)	*p*-value	Model 1 OR (95%CI)	*p*-value	Model 2 OR (95%CI)	*p*-value	Model 3 OR (95%CI)	*p-*value
DI-GM	7325	0.90 (0.86–0.94)	<0.001	0.93 (0.89–0.98)	0.004	0.93 (0.89–0.98)	0.005	0.92 (0.87–0.96)	0.001
DI-GM group									
0–3	1808	1(Ref)		1(Ref)		1(Ref)		1(Ref)	
4	1798	0.96 (0.79–1.16)	0.641	1.02 (0.84–1.24)	0.838	1.02 (0.84–1.24)	0.847	0.98 (0.80–1.19)	0.815
5	1743	0.82 (0.67–1.00)	0.047	0.90 (0.73–1.10)	0.304	0.90 (0.73–1.10)	0.297	0.85 (0.69–1.05)	0.127
≥6	1976	0.66 (0.54–0.80)	<0.001	0.77 (0.62–0.95)	0.014	0.77 (0.62–0.95)	0.017	0.72 (0.58–0.90)	0.003
Trend test			<0.001		0.008		0.009		0.002
Beneficial to gut microbiota	7325	0.83 (0.79–0.88)	<0.001	0.88 (0.83–0.94)	<0.001	0.88 (0.83–0.94)	<0.001	0.89 (0.84–0.95)	0.001
Unfavorable to gut microbiota	7325	1.01 (0.94–1.08)	0.869	1.01 (0.94–1.08)	0.845	1.01 (0.94–1.08)	0.838	0.94 (0.86–1.02)	0.112

BMI, body mass index; CVD, cardiovascular diseases; DI-GM, dietary index for gut microbiota; NHANES, National Health and Nutrition Examination Survey; OR (95%CI), Odds ratios (ORs) with their 95% confidence intervals (CIs); Ref, reference. The DI-GM ranges from 0 to 13 (including beneficial to gut microbiota [ranges from 0 to 9] and unfavorable to gut microbiota [ranges from 0 to 4]) and grouped according to 0–3, 4, 5, and ≥ 6. Model 1 adjusted for socioeconomic factors (age, race/ethnicity, marital status, education level, family income, smoking status, drinking state, physical activity, and BMI). Model 2 was adjusted for model 1 + hypertension, CVD, stroke, diabetes and antidepressants. Model 3 was adjusted for model 2 + energy intake, carbohydrate intake.

### 3.4 Subgroup analyses

**Figure 2:**
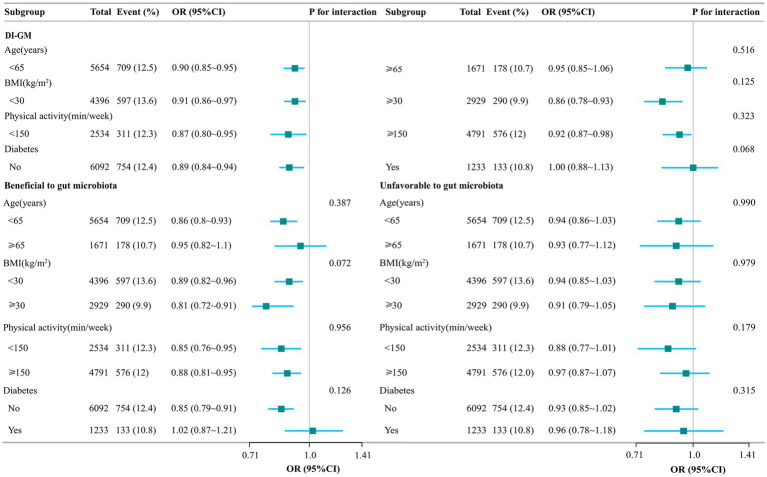
Stratified analysis of the association between DI-GM and constipation. Model adjusted for age, gender, race/ethnicity, marital status, education level, family income, smoking status, drinking state, physical activity, BMI, hypertension, CVD, stroke, diabetes, antidepressants, energy intake, and carbohydrate intake. The DI-GM ranges from 0 to 13 (including beneficial to gut microbiota [ranges from 0 to 9] and unfavorable to gut microbiota [ranges from 0 to 4]).

Subgroup analyses showed stable results between DI-GM scores and constipation (in subgroups including beneficial gut flora and unfavorable gut flora) in female participants, adjusted for age, BMI, physical activity, and diabetes, none of which were found to interact (Figure 2).

## 4 Discussion

In this study, we found that as DI-GM scores increased, the risk of constipation decreased in women, with a 28% reduction in the DI-GM ≥ 6 group compared to the DI-GM 0–3 group (OR 0.72 [95% CI 0.58–0.9]; *p* = 0.003). The beneficial gut microbiota analysis yielded similar results, with an 11% reduction in the risk of constipation (OR 0.89 [95% CI 0.84 to 0.95]; *p* = 0. 001). Subgroup analyses showed that the association between DI-GM score and constipation remained stable.

Diet and constipation have a long history in modern society and in the female population, where different habits lead to different dietary patterns (31–33). Different foods play an important role in the development and treatment of constipation (34). Foods are beneficial not only because they are rich in dietary fiber, vitamins, polyphenols, and other active ingredients (e.g., avocados are rich in dietary fiber, chickpeas are rich in proteins and vitamins, and coffee beans contain biologically active ingredients) (35–37), but also because of the effect of the composition of the food on the intestinal flora and population. Soy, whole grains, and fermented dairy products promote the proliferation of gut flora such as Lactobacillus and Bifidobacterium. Conversely, unfavorable components of food (e.g., red meat, refined flour, and processed meats) predispose individuals to intestinal inflammation and disruption of the gut flora (38). Different dietary patterns also have different effects on the risk of developing constipation. For example, Mediterranean and high-fiber diets are associated with a lower risk of constipation than Western and ketogenic diets (39). The higher the dietary pattern of food components with beneficial intestinal flora, the higher the DIGM score and the lower the risk of constipation.

The intestinal flora is a complex ecosystem that plays an important role in maintaining intestinal function and the ecological barrier of the body (40). Previous studies have demonstrated a strong association between the development of chronic constipation and disturbances in gut microbiota composition and function, as well as related metabolic dysregulation (41, 42). Dysbiosis of the intestinal flora is characterized by a decrease in the abundance of *Bifidobacterium* spp., *Lactobacillus* spp.*, Prevotella* spp., and butyrate-producing genera and has been demonstrated in patients with chronic constipation (13). Metabolites of intestinal biota, short-chain fatty acids, and methane alter intestinal pH, 5-hydroxytryptamine release, mucin secretion, and depolarization of intestinal smooth muscle ion channels (43). The effects of intestinal colonizing bacteria and their metabolites on intestinal function are reciprocal, regulating intestinal peristalsis, transport, secretion, and osmolality through the brain-gut-microbiota axis by secreting catecholamines and serotonin (44). Increasing the species and number of beneficial intestinal flora and improving the biota and metabolism of intestinal colonizing bacteria is an important approach to treating patients with constipation. Given the correlation between diet, intestinal flora, and constipation, remodeling the structure of the intestinal flora by adjusting dietary patterns is an effective strategy for relieving or treating constipation (45). Increasing dietary fiber intake in patients with constipation is the most commonly used method. On the one hand, dietary fiber has a significant effect on altering intestinal flora, and high-fiber diets can improve the number and distribution of beneficial private and Bacteroides bacteria and maintain the diversity of intestinal flora. In contrast, dietary fiber attenuates the inflammatory response, reduces intestinal inflammation, decreases intestinal mucosal damage, and inhibits local and systemic inflammatory responses (46, 47). Probiotics are often used in the prevention and treatment of constipation; they not only further break down and digest food to provide the necessary energy for intestinal cells but also effectively stimulate intestinal peristalsis and promote defecation. Moreover, the beneficial bacteria in probiotics can compete for the survival space of harmful intestinal flora, inhibit the overgrowth of harmful bacteria, and maintain the stability of the intestinal flora (45, 48). Our study showed that an increase in DI-GM helps to reduce the risk of constipation and that a good dietary pattern has a positive effect on the maintenance of intestinal flora homeostasis.

This study has several limitations. First, the initial ‘DI-GM’ was constructed using 14 food items and the specific type of tea consumption was not recorded in the NHANES data, and thus its specific parameters could not be obtained; in the future, alternative food items for tea consumption could be sought according to the NHANES dietary categories to enhance the convincing nature of the data. Second, constipation was identified based on reduced stool frequency and stool type characteristics, in addition to other symptoms such as incomplete stools and straining to pass stools. This information could not be provided because of the lack of content in the Bowel Health Questionnaire, and further bowel questionnaire items and information collection will be conducted in the future to define constipation according to the Rome VI criteria. Third, the NHANES 24-h diet, constipation, and other covariate data were self-reported and may have recall bias. Future sensitivity analyses and propensity score matching should be used to rule out the influence of residual confounders on the results. Finally, the cross-sectional design of this study was unable to determine a causal relationship between DI-GM and constipation. Therefore, further sample size expansion, cohort studies, and randomized controlled trials are needed to clarify the relationship between GI-GM and constipation.

## 5 Conclusion

DI-GM was negatively associated with the incidence of constipation in the female population. Clinicians should consider the influence of dietary structure on the treatment of constipation in women. Dietary intervention can be an important strategy for the comprehensive treatment of constipation.

## Data Availability

The raw data supporting the conclusions of this article will be made available by the authors, without undue reservation.

## Footnotes

^1^ http://www.Rproject.org
